# Dual Bioactivities of Essential Oil Extracted from the Leaves of *Artemisia argyi* as an Antimelanogenic *versus* Antioxidant Agent and Chemical Composition Analysis by GC/MS

**DOI:** 10.3390/ijms131114679

**Published:** 2012-11-12

**Authors:** Huey-Chun Huang, Hsiao-Fen Wang, Kuang-Hway Yih, Long-Zen Chang, Tsong-Min Chang

**Affiliations:** 1 Department of Medical Laboratory Science and Biotechnology, China Medical University, No 91 Hsueh-Shih Road, Taichung 40402, Taiwan; E-Mail: lchuang@mail.cmu.edu.tw; 2 Department of Hair styling & Design, Hung Kuang University, No. 34, Chung-Chie Road, Shalu, Taichung 43302, Taiwan; E-Mail: ygl615@yahoo.com.tw; 3 Department of Applied Cosmetology & Master Program of Cosmetic Science, Hung Kuang University, No. 34, Chung-Chie Road, Shalu, Taichung 43302, Taiwan; E-Mail: khyih@sunrise.hk.edu.tw; 4 General of Agriculture Bureau of Taichung City, No. 89, Sec 2, Taichung Port Road, Xitun Dist., Taichung 40701, Taiwan; E-Mail: m35208@taichung.gov.tw

**Keywords:** *Artemisia argyi*, essential oil, tyrosinase, melanin, antioxidant

## Abstract

The study was aimed at investigating the antimelanogenic and antioxidant properties of essential oil when extracted from the leaves of *Artemisia argyi*, then analyzing the chemical composition of the essential oil. The inhibitory effect of the essential oil on melanogenesis was evaluated by a mushroom tyrosinase activity assay and B16F10 melanoma cell model. The antioxidant capacity of the essential oil was assayed by spectrophotometric analysis, and the volatile chemical composition of the essential oil was analyzed with gas chromatography-mass spectrometry (GC/MS). The results revealed that the essential oil significantly inhibits mushroom tyrosinase activity (IC_50_ = 19.16 mg/mL), down-regulates B16F10 intracellular tyrosinase activity and decreases the amount of melanin content in a dose-dependent pattern. Furthermore, the essential oil significantly scavenged 2,2-diphenyl-1-picryl-hydrazyl (DPPH) and 2,2′-azino-bis (3-ethylbenzthiazoline- 6-sulphonic acid) ABTS radicals, showed an apparent reduction power as compared with metal-ion chelating activities. The chemicals constituents in the essential oil are ether (23.66%), alcohols (16.72%), sesquiterpenes (15.21%), esters (11.78%), monoterpenes (11.63%), ketones (6.09%), aromatic compounds (5.01%), and account for a 90.10% analysis of its chemical composition. It is predicted that eucalyptol and the other constituents, except for alcohols, in the essential oil may contribute to its antioxidant activities. The results indicated that essential oil extracted from *A. argyi* leaves decreased melanin production in B16F10 cells and showed potent antioxidant activity. The essential oil can thereby be applied as an inhibitor of melanogenesis and could also act as a natural antioxidant in skin care products.

## 1. Introduction

Melanin is a dark pigment produced by epidermal melanocytes. It is responsible for skin color and also plays an important role in protecting the skin from UV light-induced damage. UV-induced skin hyperpigmentation is resulted from abnormal melanin production [1]. It is also reported that several skin hyperpigmentation disorders such as freckles, melasma, age spots, post-inflammatory melanoderma and other hyperpigmentation syndromes are the result of abnormal accumulation of melanin [2]. In the first two steps of melanogenesis pathway, tyrosinase is a rate-limiting enzyme involved in hydroxylation of l-tyrosine to l-3,4-dihydroxyphenylalanine (l-DOPA), and l-DOPA is further oxidized to the corresponding *o*-quinone [3]. Hence, many skin depigmenting chemicals such as kojic acid [4], arbutin [5] and azelaic acid [6], which act as tyrosinase inhibitors, have been applied in skin whitening products for the treatment or prevention of abnormal skin pigmentation [7]. However, various chemical depigmenting agents show side effects such as the genotoxic effect of arbutin [8] and pigmented contact dermatitis due to kojic acid [9]; furthermore, transient erythema and skin irritation caused by azelaic acid [10] have been reported. Hence, searching for a safe and effective skin whitening agent is still a goal in the fields of cosmetic research and development.

Antioxidants have been intensively studied in pharmaceutical and dermatological fields as a prevention or treatment of disorders related to oxidative stress. In the past, antioxidants also have been used in the food industry to protect against the deterioration of food and in the cosmetic industry to delay or prevent skin aging. Free radicals and reactive oxygen species (ROS) are reported to be associated with several diseases such as inflammation [11], aging and age-related diseases [12]. Importantly, free radical damage on the skin caused by ROS and UV-irradiation stress plays a key role in photoaging [13,14]. Antioxidants are reported to interfere with the oxidation process by scavenging free radicals and ROS or by chelating oxidation-catalytic metals [15,16]. Hence, many natural antioxidants or antioxidant supplements have been used to reduce oxidative stress or damage in the human body [17,18]. However, some synthetic chemical antioxidants, such as *tert*-butyl hydroxyanisole (BHA) and *tert*-butyl hydroxytoluene (BHT) have been shown to exhibit carcinogenic effects on human health [19]. Therefore, a lot of studies on plant-derived antioxidants have been reported over the past decade. Additionally, it is found that ROS can accelerate skin pigmentation. Among the ROS derived from keratinocytes, NO acts to induce melanogenesis by increasing the amount of tyrosinase and tyrosinase-related protein 1(TRP-1) [20,21]. The contribution of ROS to melanogenesis has been studied by using antioxidants such as *N*-acetyl cysteine to abolish UVB-induced α-Melanocyte stimulating hormone [22]. In addition, it was found that stimulation of an endogenous antioxidant, metallothionein, also suppress melanogenesis in melanocytes [23].

Furthermore, it was found that melanogenesis produces hydrogen peroxide (H_2_O_2_) and ROS which places melanocytes under high-grade oxidative stress. Importantly, it is well known that some ROS scavengers and inhibitors of ROS generation may downregulate UV-induced melanin production [24]. Therefore, inhibitors of melanogenesis, antioxidants and ROS scavengers have been increasingly applied to skin care cosmetics for the prevention of undesirable skin hyperpigmentation [25].

*Artemisia argyi* is herbaceous perennial plant and is a widely used traditional Chinese medicine. It is native to China, Japan and is also grown in many parts of Taiwan on account of its medicinal properties. *A. argyi* is reported to possess various pharmacological activities including an antimutagenic effect [26], anti-tumor activity [27–29] and inhibitory activity against the HPV oncoprotein function [30]. The biological activities of essential oils extracted from *A. argyi* leaves have been studied. For example, the essential oil from leaves of *A. argyi* is reported to show anti-histamatic effect [31] and antifungal activity [32]. Recently, the chemical composition of essential oils extracted from leaves or flowers of *A. argyi* has been reported [32,33]. However, the inhibitory action of essential oils extracted from *A. argyi* on melanogenesis has never been explored. Recently, our laboratory has focused on searching for valuable plant essential oils with dermatological usefulness [34]. In this study, we examined the inhibitory effects on melanogenesis and antioxidant capacity of essential oil extracted from leaves of *A. argyi* and analyzed its chemical composition by GC/MS. Hence, antimelanogenic *versus* antioxidant efficacy of *A. argyi* essential oil and its chemical composition are reported in the present study.

## 2. Results and Discussion

### 2.1. Inhibitory Effect of *A. argyi* Essential Oil on Mushroom Tyrosinase Activity

**Figure 1:**
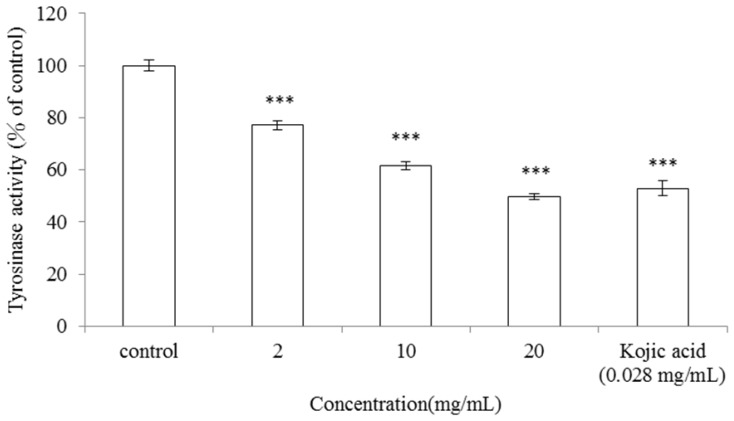
Inhibitory effect of *A. argyi* essential oil on mushroom tyrosinase activity. Different concentrations of *A. argyi* essential oil (2, 10, 20 mg/mL) or kojic acid (0.028 mg/mL) were incubated with the same units of mushroom tyrosinase. Results are represented as percentages of control, and data are presented as mean ± SD for three separate experiments. Values are significantly different by comparison with control. *** *p* < 0.001.

To determine the potential inhibitory effect of *A. argyi* essential oil on mushroom tyrosinase activity, enzyme inhibition experiments were done in triplicate. Kojic acid was used as a positive standard. The data indicated that mushroom tyrosinase activity was inhibited by the various concentrations of *A. argyi* essential oil (2, 10 and 20 mg/mL). The residual tyrosinase activity was 77.12% ± 1.64%, 61.49% ± 1.48% and 49.77% ± 1.14% of control, respectively (*p* < 0.001). IC_50_ of the essential oil is 19.16 mg/mL. Simultaneously, mushroom tyrosinase activity was inhibited by kojic acid (0.028 mg/mL) and the remained enzyme activity was 52.93% ± 2.82% of that of control (*p* < 0.001) (Figure 1).

Mushroom tyrosinase has been widely used as the enzyme for screening possible inhibitors of melanogenesis. The results indicated that the essential oil extracted from leaves of *A. argyi* effectively inhibited mushroom tyrosinase activity. The highest concentration of the essential oil (20 mg/mL) exhibited a similar inhibitory effect on mushroom tyrosinase activity as kojic acid does, hence *A. argyi* essential oil may act as a possible tyrosinase inhibitor. So far, there is no report about the dermatological application of essential oils extracted from plants of the *Artemisia* family. This is the first report that essential oil extracted from leaves of *A. argyi* significantly inhibits mushroom tyrosinase activity.

### 2.2. Effect of *A. argyi* Essential Oil on Melanogenesis in B16F10 Cells

**Figure 2:**
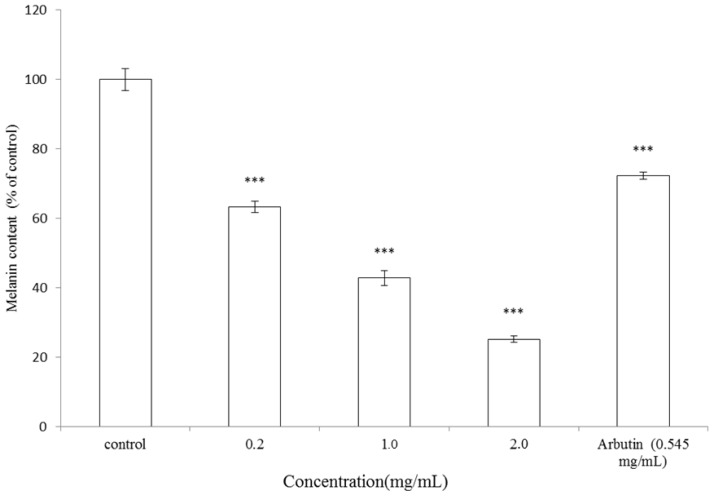
Effect of *A. argyi* essential oil on melanogenesis in B16F10 cells. Melanin content measurement was performed as briefly described below. The cells were cultured with α-MSH (100 nM) for 24 h, and then the melanin content was measured after treatment with various concentrations of *A. argyi* essential oil (0.2, 1.0 and 2.0 mg/mL) or arbutin (0.545 mg/mL) for 24 h. Results are represented as percentages of the control, and data are presented as mean ± SD for three separate experiments. Values are significantly different by comparison with control. *** *p* < 0.001.

In order to investigate the inhibitory effect of *A. argyi* essential oil on melanogenesis, the melanin content in B16F10 melanoma cells was measured after treatment with various concentrations of the essential oil. B16F10 cells were first stimulated with α-melanocyte stimulating hormone (α-MSH) for 24 h and then cultured in the presence of the essential oil at 0.2, 1.0 and 2.0 mg/mL or arbutin (0.545 mg/mL) for another 24 h. Treatment with *A. argyi* essential oil showed a significant inhibitory effect on melanin synthesis in a dose-dependent pattern. The melanin content was represented as a percentage of control. After treatment, the melanin content was 63.27% ± 1.16%, 42.84% ± 2.09% and 25.19% ± 0.98% for 0.2, 1.0 and 2.0 mg/mL of the essential oil, respectively (*p* < 0.001). IC_50_ of the essential oil is 0.769 mg/mL. Meanwhile, arbutin (0.545 mg/mL) was used as a positive standard and the residual intracellular melanin content after arbutin treatment was 72.31% ± 1.03% of control (*p* < 0.001) (Figure 2). The results shown in Figure 2 indicated that essential oil extracted from leaves of *A. argyi* exhibit a stronger inhibitory effect on melanin formation in B16F10 cells than arbutin.

### 2.3. Inhibitory Effect of *A. argyi* Essential Oil on Intracellular Tyrosinase Activity in B16F10 Cells

**Figure 3:**
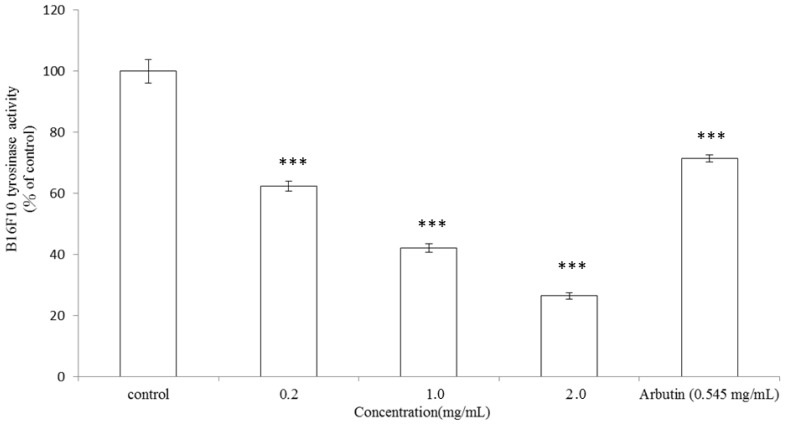
Inhibitory effect of *A. argyi* essential oil on intracellular tyrosinase activity in B16F10 cells. B16F10 cells were stimulated with α-MSH (100 nM) for 24 h, and the cellular tyrosinase activity was assayed after treatment with *A. argyi* essential oil (0.2, 1.0 and 2.0 mg/mL) or arbutin (0.545 mg/mL). Results are represented as percentages of control, and the data are mean ± SD for three separate experiments. Values are significantly different by comparison with control. *** *p* < 0.001.

To further examine the action mechanism of the inhibitory effect of *A. argyi* essential oil on melanogenesis, we assessed intracellular tyrosinase activity in B16F10 cells after treatment with the essential oil. The cells were stimulated with α-MSH (100 nm) for 24 h, and with various concentrations of the essential oil at 0.2, 1.0 and 2.0 mg/mL or arbutin (0.545 mg/mL) for another 24 h. The essential oil significantly inhibited α-MSH-induced tyrosinase activity in a dose-dependent pattern (Figure 3). After these treatments, the remaining intracellular tyrosinase activity was 62.36% ± 1.72%, 42.09% ± 1.35% and 26.42% ± 0.95% for 0.2, 1.0 and 2.0 mg/mL of the essential oil, respectively (*p* < 0.001). IC_50_ of the essential oil is 0.744 mg/mL. On the other hand, the intracellular tyrosinase activity was 71.4% ± 1.13% after arbutin treatment (*p* < 0.001). The results shown in Figure 3 were in accordance with the results indicated in Figure 2, which implies that essential oil extracted from leaves of *A. argyi* inhibited B16F10 intracellular tyrosinase activity and then decreased the melanin content in a dose-dependent manner. The action concentration of arbutin (0.545 mg/mL) is equivalent to 2.0 mM, which is often used as a standard concentration in assays of cellular tyrosinase and melanin content.

It should be emphasized that there is no report about dermatological application of essential oil extracted from leaves of *A. argyi*. Furthermore, the present study proved that *A. argyi* essential oil shows considerable depigmentation potential in the B16F10 cell model. The results shown in Figure 2 indicated that *A. argyi* essential oil has a stronger inhibitory effect on melanin synthesis in B16F10 cells than arbutin. Additionally, the essential oil also inhibited α-MSH-induced intracellular tyrosinase activity in a dose-dependent pattern (Figure 3). In these experiments, α-MSH acted as a cyclic-3′,5′-adenosine monophosphate (cAMP) inducer to stimulate melanin synthesis. It is reported that α-MSH can bind melanocortin 1 receptor (MC1R) and activate adenylate cyclase, which in turn transforms ATP to cAMP, thus increasing intracellular cAMP levels [35]. In the present study, the results evidenced that *A. argyi* essential oil inhibited B16F10 melanogenesis induced by α-MSH mediated intracellular cAMP up-regulation.

### 2.4. DPPH Scavenging Capacity Assay

**Figure 4:**
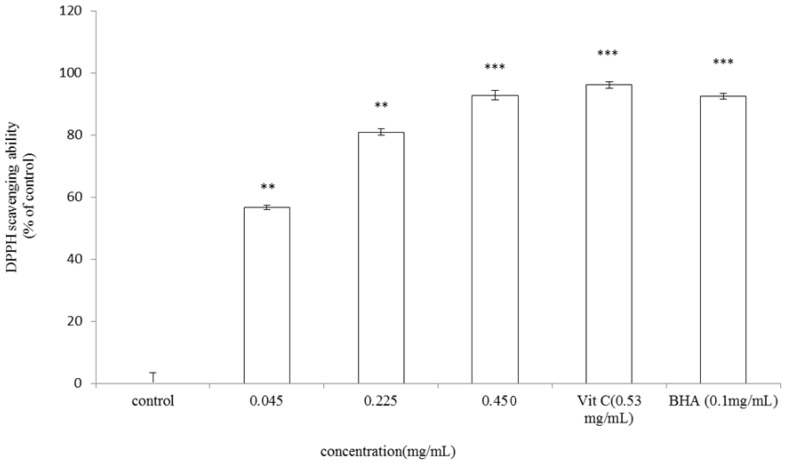
DPPH radical scavenging capacity of *A. argyi* essential oil. The essential oil at various concentrations (0.045, 0.225, 0.450 mg/mL), vitamin C (0.53 mg/mL) or BHA (0.1 mg/mL) was interacted with DPPH, respectively. The control indicated DPPH only. Results are represented as percentages of control, and the data are mean ± SD for three separate experiments. Values are significantly different by comparison with control. ** *p* < 0.01, *** *p* < 0.001.

The antioxidant capacity of *A. argyi* essential oil was first determined by measuring its DPPH scavenging ability. The *A. argyi* essential oil showed DPPH radical scavenging activity in a dose-dependent pattern as shown in Figure 4. DPPH scavenging activity of 0.045, 0.225 and 0.450 mg/mL of the essential oil was 56.67% ± 0.66%, 80.89% ± 1.06% and 92.79% ± 1.56% of control, respectively. The activity of the essential oil is slightly less effective than that of vitamin C (96.09% ± 1.01%), but is similar to that of BHA (92.41% ± 0.95%). DPPH assay is a common method to give reliable information concerning the antioxidant activity of specific compounds or extracts across a short time scale. Our results indicated that essential oil extracted from the leaves of *A. argyi* exhibit DPPH free radical scavenging activity and therefore could be applied as an antioxidant agent.

### 2.5. ABTS Scavenging Ability Assay

**Figure 5:**
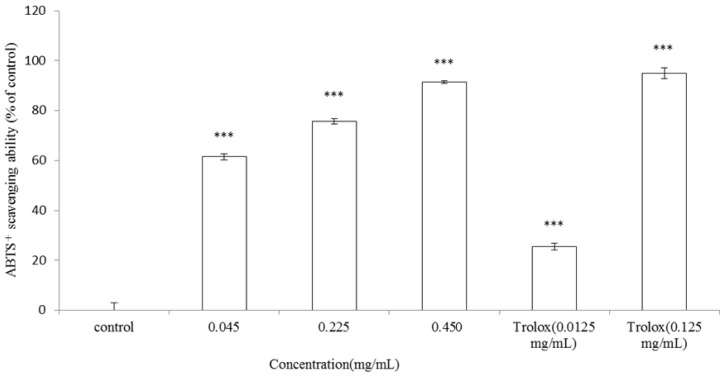
ABTS^+^ radical scavenging ability of *A. argyi* essential oil. The essential oil (0.045, 0.225 and 0.450 mg/mL) or Trolox^®^ (0.0125 or 0.125 mg/mL) were incubated with ABTS. The control indicated ABTS only. Results are represented as percentages of control, and the data are mean ± SD for three separate experiments. Values are significantly different by comparison with control. *** *p* < 0.001.

The ABTS^+^ assay was further employed to confirm the antioxidant activity of the essential oil. Different concentrations of the *A. argyi* essential oil (0.045, 0.225 and 0.450 mg/mL) or Trolox^®^ (0.0125 or 0.125 mg/mL) were incubated with ABTS^+^ solution, respectively. The ABTS^+^ scavenging capacity of the essential oil was 61.49% ± 1.12%, 75.7% ± 1.16% and 91.41% ± 0.57% of control for the essential oil at the dosage of 0.045, 0.225 and 0.450 mg/mL, respectively (*p* < 0.001). Meanwhile, the ABTS^+^ scavenging capacity of Trolox^®^ (0.0125 mg/mL) was 25.49% ± 1.31% (*p* < 0.001). The results indicated that essential oil extracted from the leaves of *A. argyi* scavenged ABTS^+^ radicals significantly in a dose-dependent pattern. However, the higher concentration of Trolox^®^ (0.125 mg/mL) still showed the strongest ABTS^+^ radical scavenging capacity (94.97% ± 2.15%) (*p* < 0.001) (Figure 5).

### 2.6. Determination of Reducing Power of *A. argyi* Essential Oil

**Figure 6:**
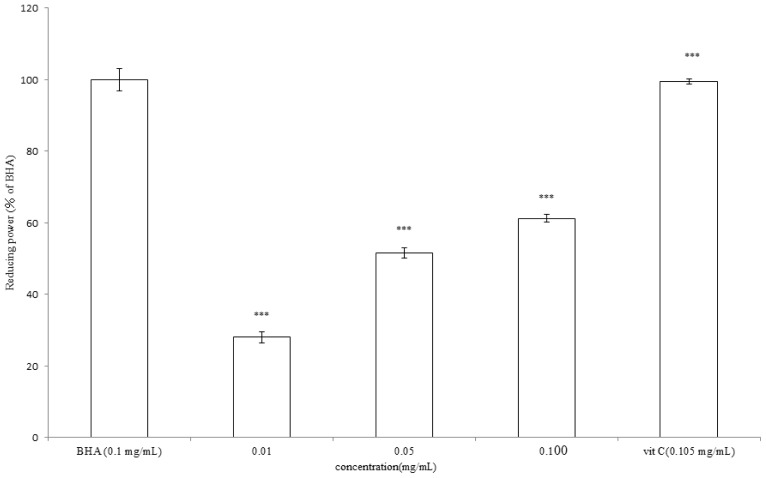
Reducing power of *A. argyi* essential oil. Different concentrations of *A. argyi* essential oil (0.01, 0.05, 0.100 mg/mL), vitamin C (0.105 mg/mL) or *tert*-butyl hydroxyanisole (BHA) (0.1 mg/mL) were used in the study. Results are represented as percentages of control, and the data are mean ± SD for three separate experiments. Values are significantly different by comparison with control. *** *p* < 0.001.

To measure the reducing power of *A. argyi* essential oil, various concentrations of the essential oil or vitamin C (0.105 mg/mL) or BHA (0.1 mg/mL) were tested according to the method of Oyaizu with a slight modification [36]. The results shown in Figure 6 revealed that higher concentrations of the essential oil present apparent reducing power. The reducing power of 0.01, 0.05, 0.100 mg/mL of *A. argyi* essential oil was 28.05% ± 1.52%, 51.68% ± 1.44% and 61.26% ± 1.09% when compared to 0.1 mg/mL of BHA (*p* < 0.001). Even though increases the dosage, the reducing power of the essential oil was still lower than those of vitamin C. Besides, the reducing power of vitamin C was almost equivalent to that of BHA.

### 2.7. Metal-ion Chelating Activity of *A. argyi* Essential Oil

**Figure 7:**
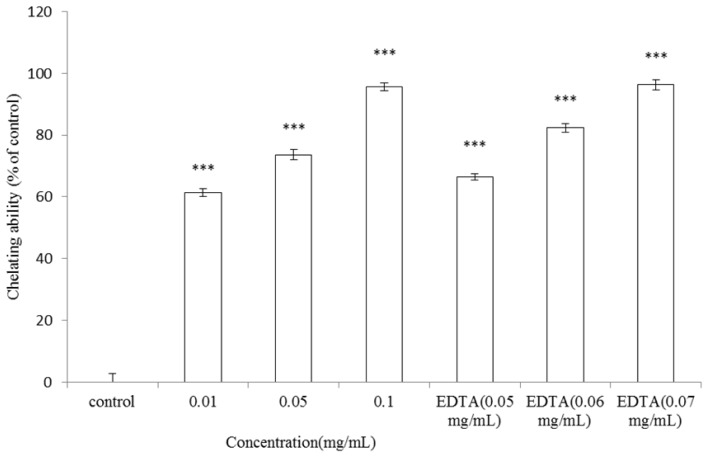
Metal-ion chelating activity of *A. argyi* essential oil. Different concentrations of *A. argyi* essential oil (0.01, 0.05, and 0.1 mg/mL) or EDTA (0.05, 0.06 and 0.07 mg/mL) were used in the study. The control indicated metal-ion solution only. Results are represented as percentages of control, and the data are mean ± SD for three separate experiments. Values are significantly different by comparison with control. *** *p* < 0.001.

The metal-ion chelating ability of 0.01, 0.05, and 0.1 mg/mL of *A. argyi* essential oil was 61.37% ± 1.19%, 73.65% ± 1.75% and 95.63% ± 1.19% of control, respectively (*p* < 0.001). On the other hand, the metal-ion chelating capacity of 0.05, 0.06 and 0.07 mg/mL of EDTA (ethylene diamine-*N*,*N*-tetraacetic acid) were 66.51% ± 1.02%, 82.30% ± 1.49% and 96.22% ± 1.70%, respectively (*p* < 0.001) (Figure 7). Antioxidants may interact with ferrous ions to form insoluble metal complexes and then inhibit interaction between metal and lipid. The results shown in Figure 7 indicated that the reducing power of 0.1 mg/mL essential oil is equivalent to that of 0.07 mg/mL of EDTA. It was further confirmed that *A. argyi* essential oil has potent antioxidant capacity.

Human skin is exposed to UV light or environmental oxidizing pollutants and becomes a preferred target of oxidative stress. It is evidenced that UV irradiation induces ROS generation in cutaneous tissue provoking damages such as enzyme inactivation and lipid peroxidation [37]. In order to counteract the oxidative damage, skin is equipped with a network of enzymatic and non-enzymatic antioxidant systems [38]. To elucidate the antioxidant activity of *A. argyi* essential oil, DPPH, ABTS^+^ radical scavenging activity, reducing power and metal-chelating capacity of the essential oil were determined as previously described [36,39]. *A. argyi* essential oil showed considerable antioxidant potential in all the above analytical studies. The results proved the antioxidant potential of *A. argyi* essential oil over different ranges with distinct efficiencies. The differential free radical scavenging activities of the essential oil against DPPH and ABTS^+^ radicals may be resulted from different mechanisms of the antioxidant-radical interactions in the two assays. Besides, the stoichiometry of reactions between the potential antioxidant chemicals in the essential oil may be different, which result in the difference in radical scavenging capacity [40]. The reducing power of the antioxidant converts a Fe^3+^/ferricyanide complex to the ferrous form and can serve as an indicator of the antioxidant capacity. The existence of reductones accounts for the reducing power and exhibits the antioxidant activities by donating a hydrogen atom. The percentage of ketone content in the essential oil is 6.09%, which may account for its reducing power. Antioxidant may form insoluble metal complexes with ferrous ion and then inhibit interaction between metal and lipid. The higher metal-ion chelating capacity of the essential oil indicated its potent antioxidant activity (Figure 7). It is reported that various metal chelators such as kojic acid [41] and *N*-phenylthiourea (PTU) [42] act as tyrosinase inhibitors because the enzyme has a dinuclear copper in its active site.

The idea behind searching antioxidants for skin-whitening efficacies lies in the hypothesis that the oxidative stress resulted from UV-irradiation may contribute to stimulation of melanogenesis. It was reported that UV irradiation can produce ROS in the cutaneous tissues that may induce melanogenesis by activating tyrosinase as the enzyme prefers superoxide anion radical (O_2_^−^) over oxygen molecule (O_2_) [43]. Additionally, it was found that redox agents can also influence melanin production by interacting with copper ion at the active site of tyrosinase or with *o*-quinones to block the oxidative polymerization of melanin intermediates [44]. Moreover, antioxidants such as vitamin B, vitamin C or vitamin E can also reduce the photooxidation of pre-existing melanin particles. Hence, these vitamins with antioxidant activity are common applied in skin-whitening cosmetic formulations [45]. In addition, our research showed the apparent antioxidant capacities of *A. argyi* essential oil also indicated it could be applied into formulation of skin care cosmetic products.

### 2.8. Chemical Composition of *A. argyi* Essential Oil

The chemical composition of *A. argyi* essential oil was analyzed by GC/MS as shown in Table 1. The major component in the oil is eucalyptol (23.66%). There are 16.72% of alcohols in the oil including (−)-borneol (6.55%), linalool (3.31%), longiborneol (2.67%), (−)-terpinen-4-ol (1.40%), (−)-menthol (1.19%), nerolidol (0.88%), dihydrocarveol (0.55%) and α-terpieol (0.17%). The sesquiterpenes in the essential oil are caryophyllene (10.19%), humulene (1.40%), β-cubebene (1.29%), α-gurjunene (0.74%), (*Z*)-β-farnesene (0.59%), δ-cadinene (0.59%) and α-bergamotene (0.41%). The oil contains six types of esters including bergamiol (3.91%), bornyl acetate (3.63%), cedryl acetate (2.28%), nerol acetate (0.99%), geranyl acetate (0.88%) and geranyl isovalerate (0.1%). There are six types of monoterpenes in the essential oil such as (−)-β-pinene (5.62%), 3-carene (4.64%), camphene (0.48%), τ-terpinene (0.36%), 1*R*-α-pinene (0.34%) and myrcene (0.19%). Menthone (4.18%), camphor (1.53%), 3-octanone (0.25%) and (+)-carvone (0.13%) are four ketones existed in the oil. The only aromatic compound in the oil was *o*-cymene (5.01%). However, there are 9.90% of unknown compounds in the essential oil.

The GC/MS data revealed the presence of seven classes of chemical components in *A. argyi* essential oil, which are summarized in Table 1, including ethers (23.66%), alcohols (16.72%), sesquiterpenes (15.21%), esters (11.78%), monoterpenes (11.63%), ketones (6.09%) and aromatic compounds (5.01%), which account for 90.10% of the essential oil. Recently, an earlier report studied volatiles from leaves and flowers of *A. argyi*. revealed that the presence of borneol and bornyl acetate existed in the two parts of the plant [33]. In the present study, we also found (−)-borneol (6.55%) and bornyl acetate (3.63%) in *A. argyi* essential oil. There are several factors involved in the regulation of the constituents in the essential oil such as different plant cultivation and/or harvesting procedures. Furthermore, different analytical technique may also result in different GC/MS data. Eucalyptol, also named as 1,8-cineole, is the major and only ether component in the essential oil. Since some synthetic ethers have been reported to show antioxidant activities [46], we hypothesized that eucalyptol may account for the antioxidant activities of the essential oil. Additionally, the other components such as sesquiterpenes, esters [47], monoterpenes [48], ketones [49,50] and aromatic compound [51] may also contribute the antioxidant activities of the essential oil extracted from the leaves of *A. argyi*.

It was found that the secondary metabolites and bioactive phytoconstituents identified by GC/MS in different plants have been previously reported which show antimicrobial, anti-inflammatory and antioxidant activities [52–55]. Further, we have screened over thirty types of plants and evaluated the potential dermatological effects of the essential oils extracted from these plants, and found the essential oils isolated from the leaves of *Vitex negundo* Linn and *Acorus macrospadiceus* (Yamamoto, F. N. Wei, Y. K. Li *et al*.) exhibit antioxidant activities and antimelanogenic properties [34,56]. Therefore, the chemical constituents found in *Artemisia argyi* leaves may play major roles in the biological activities and pharmacological properties, but the biological roles of the chemicals in both essential oils still remained to be elucidated in the near future.

## 3. Experimental Section

### 3.1. Plant Material and Extraction of Essential Oils

The leaves of *A. argyi* were gathered during April to July in 2010, dried in a shady place and identified at Taichung District Agricultural Research and Extension Station in Taiwan. Essential oil was extracted from the leaves (2 kg) by steam hydrodistillation in a Clevenger-type apparatus at 100 °C for 2 h. The essential oil was collected in a sealed glass bottle and stored in a 4 °C refrigerator until analysis. In the present study, the essential oil was diluted with dimethyl sulfoxide (DMSO) and DMSO was used as a negative control in the following experiments. The IC_50_ is the concentration of the essential oil where the absorbance is reduced by half. The IC_50_ is calculated using Microsoft excel and linear interpolation method. Briefly, five values of concentration containing the tested values were evaluated for their activities. Raw data (relative activities) are plotted against the essential concentrations. IC_50_ values can be determined using linear interpolation method.

### 3.2. Mushroom Tyrosinase Actvity Measurement

To measure the inhibitory effects of *A. argyi* essential oil on mushroom tyrosinase, enzyme inhibition experiments were carried out in triplicate as previously described with a slight modification [57]. Briefly, 20 μL of aqueous solution of mushroom tyrosinase (200 units) was added to a 96-well microplate, in a total volume of 200 μL mixture containing 5 mM l-dopa dissolved in 50 mM phosphate buffer (pH 6.8) and *A. argyi* essential oil (2, 10 and 20 mg/mL) or kojic acid (0.028 mg/mL). The assay mixture was incubated at 37 °C for 30 min. After incubation, the amount of dopachrome produced in the reaction mixture was determined by spectrophotometric analysis of absorbance at 490 nm.

### 3.3. B16F10 Intracellular Melanin Content Measurement

B16F10 melanoma cells (ATCC CRL-6475) were cultured in DMEM with 10% fetal bovine serum (FBS; Gibco, Langley, OK, USA) and penicillin/streptomycin (100 IU/50 μg/mL) in a humidified atmosphere containing 5% CO_2_ in air at 37 °C. Intracellular melanin content was measured as previous described with some modifications [58]. The cells were treated with α-MSH (100 nM) for 24 h, and further treated with either *A. argyi* essential oil (0.2, 1.0 and 2.0 mg/mL) or arbutin (0.545 mg/mL) for another 24 h. After treatments, the cells were detached by incubation in trypsin/EDTA and subsequently centrifuged at 5000*g* for 5 min, and then the cell pellets were solubilized in 1 N NaOH at 60 °C for 60 min. The melanin content was assayed at 405 nm absorbance by spectrophotometric analysis.

### 3.4. B16F10 Intracellular Tyrosinase Activity Assay

B16F10 intracellular tyrosinase activity was determined as described previously with minor modifications [59]. The cells were treated with α-MSH (100 nM) for 24 h, and then further treated with various concentrations of *A. argyi* essential oil (0.2, 1.0 and 2.0 mg/mL) or arbutin (0.545 mg/mL) for another 24 h. After treatments, the cells were washed twice with phosphate-buffered saline and homogenized with 50 mM PBS (pH 7.5) buffer containing 1.0% Triton X-100 and 0.1 mM phenylmethyl sulfonyl fluoride (PMSF). Cellular extracts (100 μL) were mixed with freshly prepared l-dopa solution (5.0 mM in 50 mM phosphate-buffered saline, pH 6.8) and incubated at 37 °C for 30 min. The absorbance at 490 nm was measured with a microplate reader Gen 5™ (BIO-TEK Instrument, Winooski, VT, USA) to monitor the production of dopachrome.

### 3.5. DPPH Scavenging Activity Assay

The antioxidant activity of *A. argyi* essential oil was first determined by measuring the DPPH scavenging ability [60,61]. The essential oil at various concentrations (0.045, 0.225 and 0.45 mg/mL) was added to 2.9 mL of DPPH (60 μM) solution. When DPPH reacts with any antioxidant in the essential oil that can donate hydrogen, it gets reduced form and the resulting decrease in absorbance at 517 nm was recorded using a UV-Vis spectrophotometer (Jasco, V-630, Tokyo, Japan). In this study, vitamin C (0.53 mg/mL) and BHA (0.1 mg/mL) were used as antioxidant standards.

### 3.6. ABTS^+^ Scavenging Capacity Assay

The ABTS decolorisation assays were carried out as previously described [62]. It involves the generation of ABTS^+^ chromophore by oxidation of ABTS with potassium persulfate. The ABTS radical cation (ABTS^+^) was produced by reacting 7 mM stock solution of ABTS with 2.45 mM potassium persulfate and allowing the mixture to stand in the dark for at least 6 h before use. Absorbance at 734 nm was measured 10 min after mixing of different concentrations of the *A. argyi* essential oil (0.045, 0.225 and 0.45 mg/mL) with 1 mL of ABTS^+^ solution. The ABTS^+^ scavenging capacity of *A. argyi* essential oil was compared with that of Trolox^®^ (0.0125 or 0.125 mg/mL).

### 3.7. Determination of Reducing Power

The reducing power of the essential oil was determined according to the method previously described by Oyaizu [36]. Different concentrations of *A. argyi* essential oil (0.01, 0.05, 0.1 mg/mL), vitamin C (0.105 mg/mL) or BHA (0.1 mg/mL) was mixed with phosphate buffer (2.5 mL, 0.2 M, pH 6.6) and potassium ferricyanide [K_3_Fe(CN)_6_] (2.5 mL, 1% *w*/*v*). The mixture was incubated at 50 °C for 20 min. A portion (2.5 mL) of trichloroacetic acid (10% *w*/*v*) was added to the mixture, which was then centrifuged at 1000*g* for 10 min. The upper layer of solution (2.5 mL) was mixed with distilled water (2.5 mL) and FeCl_3_ (0.5 mL, 0.1% *w*/*v*), and the absorbance was measured at 700 nm in a UV-Vis spectrophotometer. Higher absorbance of the reaction mixture indicated greater reducing power of the test sample.

### 3.8. Measurement of Metal-Ion Chelating Capacity

The chelation of ferrous ions by the *A. argyi* essential oil was determined as the previous method with slight modifications [39]. Different concentrations of essential oil (0.01, 0.05 and 0.1 mg/mL) or the positive standard EDTA (0.05, 0.06 and 0.07 mg/mL) were added to a solution of 1 mM FeCl_2_ (0.05 mL). Then 0.1 mL of ferrozine (1 mM) was added to the reaction mixture and the mixture was quantified to 1 mL with methanol, left standing at 25 °C for 10 min. The absorbance of the reaction mixture was measured at 562 nm. The percentage of chelating capacity of test sample was calculated as follows:

(1) $$\text{chelating capacity}\%=[(A_{1}-A_{2})/A_{1}\times 100]$$

where *A*_1_ is the absorbance of control and *A*_2_ is the absorbance in the presence of essential oil or EDTA.

### 3.9. Gas Chromatography-Mass Spectrometry (GC/MS)

Chemical components analysis of the volatiles in the *A. argyi* essential oil was carried out using a Thermo GC/MS system (GC/MS Trace DSQ-Mass Spectrometer, MSD 201351, Thermo, Minneapolis, MN, USA). The Equity-5 capillary column (Supelco, St. Louis, MO, USA) with 30 m length and 0.25 mm inside diameter was used with a 0.25 μm thick film. The oven temperature gradient was programmed as follows: isothermal at 40 °C, followed by a 5 °C temperature ramp every minute to 100 °C, which was held for 5 min. Subsequently, the temperature was increased 5 °C every minute to 250 °C and stayed for 20 min. The carrier gas was helium (1 mL/min). The temperature of injection port and detector was 250 °C. Ionization of the test essential oil (1 μL) was performed in the EI mode (70 eV). The linear retention indices for all compounds were determined by co-injection of the essential oil with a solution containing a homologous series of C8–C22 *n*-alkanes [63]. The individual components were identified by retention indices and compared with compounds known from the literature [64]. The mass spectra were also compared with known, previously obtained, compounds or from the Trace DSQ-MASS spectral database (Thermo, New York, NY, USA).

### 3.10. Statistical Analysis

Statistical analysis of the experimental data points was performed by the ANOVA test, which was used for comparison of measured data using SPSS 12.0 statistical software (SPSS Inc.: Chicago, IL, USA). Differences were considered as statistically significant at *p* < 0.05.

## 4. Conclusions

This is the first report on the inhibitory effect of essential oil as extracted from the leaves of *A. argyi* and its melanin production. We also analyzed the chemical composition and antioxidant capacities of the essential oil. The present study concludes that *A. argyi* essential oil shows antioxidant potential, which simultaneously inhibits melanin synthesis in B16F10 melanoma cells. The results indicated that *A. argyi* essential oil decreased melanin production might be attributed to its inhibitory action upon the signaling pathway regulating tyrosinase activity or depletion of cellular oxidative stress. The essential oil can thereby serve as a natural antioxidant, which could also inhibit melanin production. Our research shows that essential oils extracted from leaves of *A. argyi* could be applied into the cosmetic formulations of skin-whitening products.

## Figures and Tables

**Table 1:** Chemical composition of essential oil from leaves of *A. argyi*. The chemical constituents of *A. argyi* essential oil were analyzed by a Thermo GC/MS system.

*R*_t_^a^	Compound b	M. f. c	Peak area (%)	Classification
20.22	3-Carene	C_10_H_16_	4.64	monoterpene
21.52	Camphene	C_10_H_16_	0.48	monoterpene
24.03	(−)-β-Pinene	C_10_H_16_	5.62	monoterpene
25.35	3-Octanone	C_8_H_16_O	0.25	ketone
25.54	Myrcene	C_10_H_16_	0.19	monoterpene
26.82	Dihydrocarveol	C_10_H_18_O	0.55	alcohol
27.25	*o*-Cymene	C_10_H_14_	5.01	aromatic compound
27.55	Eucalyptol	C_10_H_18_O	23.66	ether
28.18	1R-α-Pinene	C_10_H_16_	0.34	monoterpene
29.04	τ-Terpinene	C_10_H_16_	0.36	monoterpene
30.94	Linalool	C_10_H_18_O	3.31	alcohol
32.59	Camphor	C_10_H_16_O	1.53	ketone
33.03	Menthone	C_10_H_18_O	4.18	ketone
33.58	(−)-Borneol	C_10_H_18_O	6.55	alcohol
33.97	(−)-Menthol	C_10_H_20_O	1.19	alcohol
34.15	(−)-Terpinen-4-ol	C_10_H_18_O	1.40	alcohol
34.89	α-Terpieol	C_10_H_18_O	0.17	alcohol
38.05	(+)-Carvone	C_10_H_14_O	0.13	ketone
39.07	Bergamiol	C_12_H_20_O_2_	3.91	ester
41.31	Bornyl acetate	C_12_H_20_O_2_	3.62	ester
44.59	α-Gurjunene	C_15_H_24_	0.74	Sesquiterpene
45.56	Nerol acetate	C_12_H_20_O_2_	0.99	ester
46.93	Cedryl acetate	C_17_H_28_O_2_	2.28	ester
47.48	Geranyl acetate	C_12_H_20_O_2_	0.88	ester
48.83	Caryophyllene	C_15_H_24_	10.19	Sesquiterpene
49.54	α-Bergamotene	C_15_H_24_	0.41	Sesquiterpene
50.15	Humulene	C_15_H_24_	1.40	Sesquiterpene
50.37	(*Z*)-β-Farnesene	C_15_H_24_	0.59	Sesquiterpene
51.15	β-Cubebene	C_15_H_24_	1.29	Sesquiterpene
51.68	Geranyl isovalerate	C_15_H_26_O_2_	0.1	ester
52.11	Nerolidol	C_15_H_26_O	0.88	alcohol
52.55	δ-Cadinene	C_15_H_24_	0.59	Sesquiterpene
54.29	Longiborneol	C_15_H_26_O	2.67	alcohol
	Unknown		9.90	

a R_t_: Retention time (min);

b The components were identified by their mass spectra and retention indices (RIs) with that of the Wiley and NIST mass spectral databases and the previously published RIs;

c M. f.: Molecular formula.
